# Supplementary material for: Smooth Interpolating Curves with Local Control and Monotone Alternating Curvature
Source: Comput Graph Forum. 2022 Oct 6;41(5):25–38. doi: 10.1111/cgf.14600 (PMC9827861; doi:10.1111/cgf.14600)
Supplement: Supplementary file 1 — Supplement Material [file CGF-41-25-s001.zip › Local-Smooth-Interpolating-MonoCurvature/extern/clothoids/submodules/Utils/ThirdParties/fmt/fmt-8.1.1.zip!/fmt-8.1.1/doc/html/contents.html]

Contents — fmt 8.1.1 documentation


Toggle navigation

{fmt}

- 8.1.1
  - 8.1.1
  - 8.1.0
  - 8.0.1
- Contents
  (current)
- Usage
- API
- Syntax

# Contents¶

- Usage
  - Building the Library
  - Installing the Library
  - Usage with CMake
  - Usage with build2
  - Building the Documentation
  - Conda
  - Vcpkg
  - LHelper
  - Android NDK
  - Homebrew
- API Reference
  - Core API
  - Format API
  - Ranges and Tuple Formatting
  - Date and Time Formatting
  - Format string compilation
  - Terminal color and text style
  - System APIs
  - `std::ostream` Support
  - `printf` Formatting
  - `wchar_t` Support
  - Compatibility with C++20 `std::format`
- Format String Syntax
  - Format Specification Mini-Language
  - Chrono Format Specifications
  - Format Examples

© Copyright 2012-present, Victor Zverovich.
Created using Sphinx 3.3.0.
